# Some Additional Data That Might Be Useful for Diastasis Recti Assessment

**DOI:** 10.3389/jaws.2023.10923

**Published:** 2023-02-02

**Authors:** P. Ngo, J.-P. Cossa, S. Gueroult, D. Blum, E. Pélissier

**Affiliations:** ^1^ Institut de la Hernie, Paris, France; ^2^ Établissement Français du Sang de Franche Comté, Besançon, France

**Keywords:** diastasis recti, medial bulging, global bulging, width of divarication, diastasis recti classification

## Abstract

**Background:** Diastasis recti (DR) is characterized by separation of both rectus muscles and protrusion of the median bulging, but besides median bulging DR can also entail global abdominal bulging. On other note, DR classification is based on the width of divarication, but measurement values are different at rest and at effort due to muscle contraction. Aim of the study is to provide additional features concerning the type of bulging and the width of divarication.

**Methods:** Findings were retrospectively drawn from the data prospectively collected in the records of a continuous cohort of 105 patients (89 females, 16 males) referred for diastasis and concomitant ventral hernia repair.

**Results:** There was a median bulging alone in 45 (42.9%) cases, a global bulging alone in 18 (17.1%) cases, both types combined in 37 (35.2%) cases and no bulging in 5 (4.8%). On 55 patients with a global bulging, 51 were females. Tape measurements values of DR width were closer to the values measured on the CT scan at leg raise than at rest. The differences were significant at rest as well as at leg raise. Though the difference at rest was highly significant (*p* = 0.000), the difference at effort was not far from being not significant (*p* = 0.049).

**Conclusion:** Besides median bulging, presence or absence of the global bulging should be included in DR assessment. The difference between width of divarication at rest and on exertion raises the question of which value should be used for DR classification. The question is worth being debated.

## Introduction

According to the European Hernia Society (EHS) guidelines on management of diastasis recti (DR), the latter is defined as an inter-rectus distance (IRD) exceeding 2 cm, and it is classified D1 for IRD ≥2–3 cm, D2 for IRD ≥3–5 cm and D3 for IRD ≥5 cm (1).

In most cases DR predominates at the supra and peri-umbilical parts of abdominal wall because the linea alba is in the shape of a tuning fork. Depending on the lengthwise extension, DR can be divided in three main features: supra-umbilical, supra and peri-umbilical, and supra and infra-umbilical.

**FIGURE 1:**
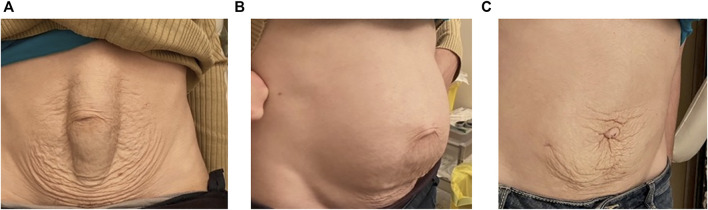
Medial bulging **(A)**, global bulging **(B)** and postoperative result **(C)** in the same patient.

Width of the divarication can be assessed by clinical examination using a tape or a caliper, as well as by ultrasound or CT scan or even MRI. According to EHS guidelines, measurement of IRD using either ultrasound or calipers is a reliable method, but there is no clear answer as to whether IRD should be measured at rest or during active muscle contraction. Since most papers providing IRD values do not specify the conditions of measuring, it is likely that they are tacitly measured at rest. However, IRD values differ depending on whether the muscles are contracted or not, since muscle contraction tends to approximate the muscle borders. Furthermore, the daily practice shows that besides the median bulging, DR can also entail global bulging of the abdominal wall, which can affect the patient’s body image (Figure 1).

Aim of the study is to provide some additional features concerning measurement of the width of divarication and the type of bulging, drawn from the data that were systematically and prospectively collected on a continuous cohort of patients referred to our institute for diastasis and ventral hernia repair.

## Patients and Methods

Data on patient demographics, as well as diastasis and hernia characteristics, are prospectively recorded in our database, with the patient consent, for every patient referred to our institute for surgery. The study is based on the retrospective review of the records.

IRB advice was not requested because all patients referred to our institution are informed that data are anonymously collected for evaluation, and the study was based on data only, not on persons or on surgical practice.

**FIGURE 2:**
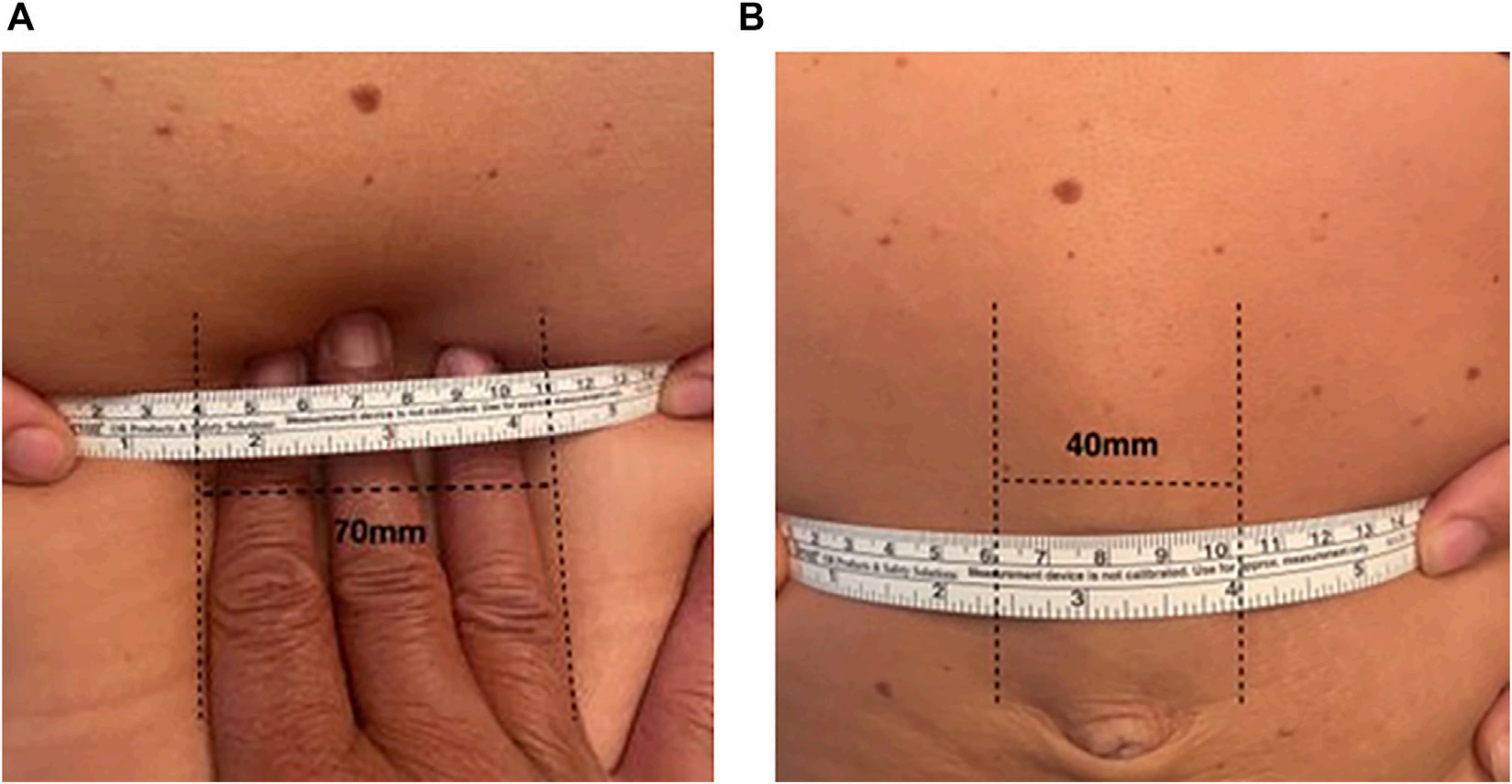
Measuring the width of divarication with a tape at rest **(A)** and at straight leg raise **(B)**.

**FIGURE 3:**
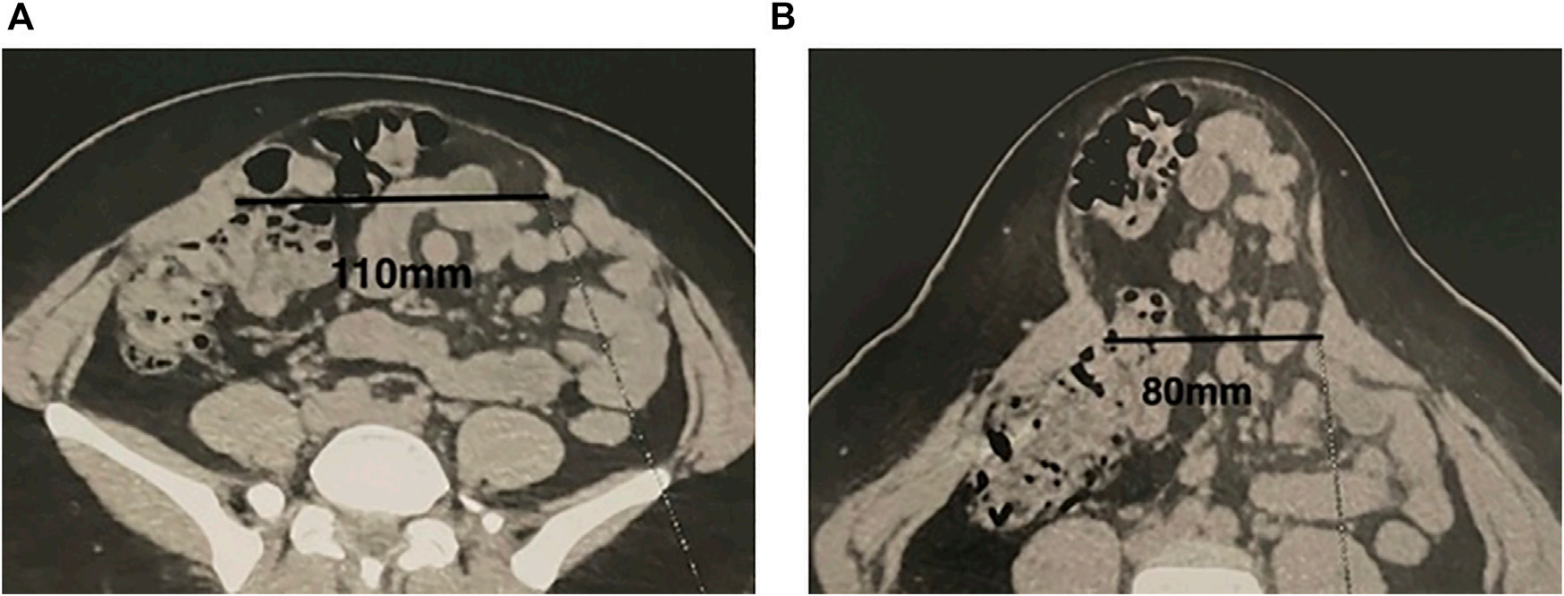
Measuring the width of divarication on the CT scan at rest **(A)** and at straight leg raise **(B)**.

In patients with DR, it is our current practice to record the presence or not of the medial bulging, the width of divarication by measuring the distance between the medial borders of both rectus muscles with a tape measure on the supine patient, 2–3 cm above the navel, at rest as well as at straight leg-raise (Figure 2). Similarly, the presence or not of the global bulging, defined as global enlargement of the abdomen, based on visual examination of the patient in the upright position at rest, is recorded. When a CT scan is performed the radiologist records the distance between the medial borders of both rectus muscles at L4 level at rest and at straight leg raise (Figure 3).

Comparison of values of the divarication width at rest, obtained by tape measuring *versus* CT scan, and same comparison of values obtained at straight leg raise, were carried out using the Student T test for paired samples, on 40 cases for which clinical data as well as CT scan measurements were fully documented.

## Results

The results are given in Tables 1–3. The cohort involved 105 patients, 89 females and 16 males, who were operated from January 2020 to August 2022; 83 of the 71 females were parous. The type of DR was supra-umbilical in 23 (21.9%) cases, supra and peri-umbilical in 47 (44.8%) cases and supra and infra-umbilical in 35 (33.3%) cases. There was a median bulging alone in 45 (42.9%) cases and a global bulging alone in 18 (17.1%) cases. Both types were present in 37 (35.2%) cases and in 5 (4.8%) cases there was no bulging at all. On 55 patients with a global bulging, 51 were females and only four were males. A ventral hernia was present in 103 (98.1%) cases (86 umbilical, 5 epigastric, 10 combined, and 2 incisional).

**TABLE 1:** Patient demographics and diastasis characteristics.

Patients n	105
Males n (%)	16 (15.2)
Females n (%)	89 (84.8)
Age median (range)	40 (18–82)
BMI median (range)	21.54 (16.9–39.8)
Parous women n (%)	83 (79)
Diastasis	
Supra umbilical n (%)	23 (21.9)
Supra and peri-umbilical n (%)	47 (44.8)
Supra and infra-umbilical n (%)	35 (33.3)
Medial bulging alone n (%)	45 (42.9)
Global bulging alone n (%)	18 (17.1)
Both types n (%)	37 (35.2)
No bulging at all n (%)	5 (4.8)
Ventral hernias n (%)	103 (98.1)

Table 2 shows that the differences between diastasis width measured with a tape or on the CT scan were significant at rest as well as at leg raise. Nevertheless, when the difference at rest was highly significant (*p* = 0.000), the difference at effort was not far from being not significant (*p* = 0.049).

**TABLE 2:** Divarication width at rest and at leg-raise, with tape measure and CT scan.

	Tape measure	CT scan	p*
At rest (mm) mean, SD	71.5, 23.04	46.7, 13.99	0.000
Leg-raise (mm) mean, SD	35.3, 12.03	39.8, 12.79	0.049

*Student T test for paired samples.

**FIGURE 4:**
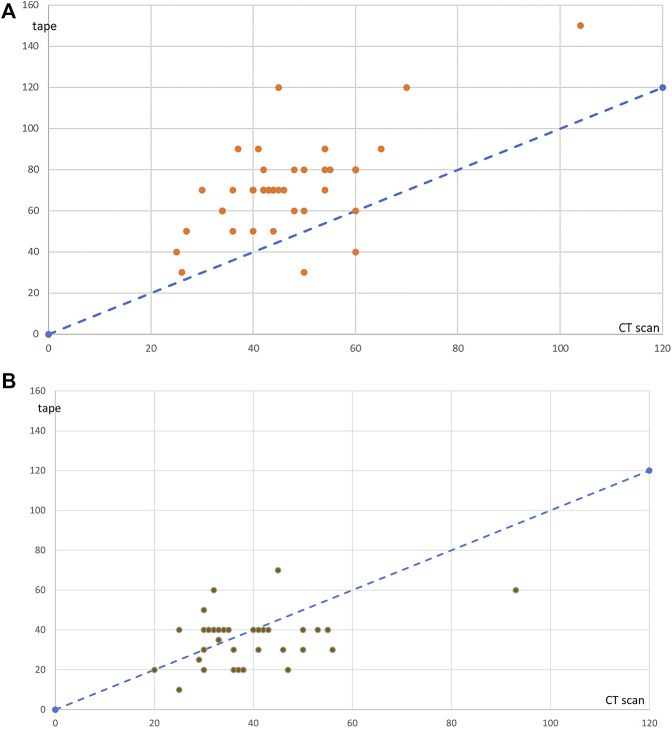
Values measured on the CT scan (x-axis) or with the tape (y-axis) for each of 40 cases, at rest **(A)** and at leg raise **(B)**. The dashed line represents the ideal position of values if tape measurements and values measured on the CT scan were identical.

Moreover, Figure 4A shows that the values obtained by tape measuring at rest are notably divergent from the dashed line and tend to overestimate the tape measurement compared to CT scan, when Figure 4B shows that values obtained by tape measuring at leg raise are close to the dashed line and homogeneously distributed on either side of the line.

These results suggest that the results obtained by tape measurement are closer to the values measured on the CT scan at leg raise than at rest.

Table 3 shows that the type of diastasis according to EHS classification would be different, depending on whether measurements are taken at rest or at leg raise.

**TABLE 3:** Width of diastasis according to EHS classification (1) at rest vs. at leg raise.

	Rest	Leg-raise
D1 ≥ 2–3 cm	0	13
D2 ≥ 3–5 cm	46	82
D3 ≥ 5 cm	59	10

## Discussion

The results highlight two main points: on one hand the median bulging was present in only 78% of the cases (47 alone and 37 combined), the global bulging was present in 52% of the cases (18 alone and 37 combined) and both were combined in 35% of the cases; on the other hand the distance between the medial borders of both rectus muscles was different at rest and at straight leg-raise and depending on whether it was measured on the patient with a ruler or on the CT scan.

The median bulging is commonly regarded as an essential characteristic of DR (2). However, in our series, the median bulging was present in only 78% of the cases, there was no bulging at all in 4.8%, and the global bulging, defined as global enlargement of the abdomen, based on the patient’s complaint and on visual examination of the patient in the upright position (Figure 1), was present in half the cases. The medial bulging is well known but the global bulging is less commonly mentioned in publications, although it can affect the patient’s body image. It is more frequent in women (51 females on 55 cases) who say, “it is as if I were pregnant”. The diagnosis of global bulging is based on visual examination of the patient in the standing position at rest, thus we suggest that examining the patient standing as well as in the lying position, be part of diastasis assessment. Measuring the waist circumference just above iliac crests might help bulging evaluation and comparison between preoperative and postoperative status. Unfortunately, we did not include this measurement in our database. Nevertheless, we suggest that measuring the waist circumference is worth being part of the checking and from now we include it in our database.

Concerning the width of divarication, the values collected at clinical examination by tape measuring and the values measured on the CT scan, were close at leg-raise, but at rest they were larger at clinical examination than on the CT scan. As a matter of fact, the difference was highly significant at rest (*p* = 0.000), but the difference at leg-raise was not far from being not significant (*p* = 0.049), as shown in Table 2. Moreover, Figure 4A shows that the values obtained by tape measuring at rest tend to overestimate the width of divarication compared to CT scan, when Figure 4B shows that the values obtained by tape measuring at leg raise are close to the values measured on the CT scan.

Differences between IRD values measured at manual examination or by imaging methods have been reported by others (3–6). However, it is difficult to know which method provides the most accurate evaluation.

According to Emanuelsson et al the CT scan underestimates IRD in comparison with tape measure at clinical examination as well as intraoperatively (3). On the contrary, Mughal and Ross consider that the right value is provided by CT scan or ultrasound (4). In the study by Nahas et al there was no difference between the values measured on the CT scan and intraoperatively (5). Chiarello et al compared IRD values measured by caliper to ultrasound at rest as well as on exertion (curl-up), they found no significant difference between both methods above the umbilicus, when IRD values measured by caliper were higher than the values measured by ultrasound below umbilicus, what suggests that in this location it is manual examination that overestimates IRD (6).

In our study comparing tape measure to CT scan at rest and at leg-raise, IRD values at rest were higher at tape measure than on the CT scan, when there was no difference between both methods at leg-raise.

Based on the above, it is difficult to conclude which is the best method to objectively assess IRD. Some explanations of these discrepancies have been suggested.

Emanuelsson et al (3) suggested two possible explanations: one is that during scanning the patients tend to involuntarily contract their abdominal muscles, what is plausible. The other one is that the CT scan identifies the most medially located muscular fibers “even though they have no relevance,” but these medial fibers are actual muscular fibers that participate to the suturing repair, and there is no reason to consider that they are negligible.

It may also be that identification of the medial bounds of rectus muscles is less precise at manual measurement, due to subcutaneous tissue thickness, as suggested by Chiarello et al. (6).

Ultrasound, which is commonly used in current practice (1, 7, 8), might be the best method, provided it is performed by an experienced operator, because it allows dynamic imaging: by talking to the patient the operator can obtain muscle relaxation as well as contraction.

If we admit that CT scan images correspond well to anatomy, our results showing that values provided by tape measurements are closer to values measured on the CT scan at leg raise than at rest, suggest that measurements on exertion should be taken in consideration.

Given the ambiguity as to the best method of IRD measurement, some other studies comparing tape measurement, caliper, intraoperative, ultrasound and CT scan, are worth being carried out.

Table 3 shows that the type of diastasis according to EHS guidelines classification (1) would be different, depending on whether measurements are taken at rest or at leg raise, whereas correct categorization of the cases has practical significance. This finding raises the question of which value should be taken in consideration for diastasis classification, namely at rest or on exertion, or both. In current practice the CT scan is indeed not routinely performed. In most cases the classification is based on clinical measurements, and classifying the cases makes practical sense, since the width of divarication can influence the choice of surgical technique.

As a matter of fact, Bellido Luque et al. (9) suggest that a diastasis greater than 6–7 cm of separation could benefit from the use of a reinforcement patch rather than a simple suture because of excessive tension on the suture line, but they do not specify if this statement is based on measurements taken at rest or on exertion. Based on our clinical experience, though not on evidence-based data, we consider that tension on the suture line is excessive when IRD is superior to 6 cm on exertion. On the contrary when IRD is superior to 6 cm at rest, the laxity of aponeurotic structures allows tension-free approximation suture of rectus muscles.

The principal strength of this study is that it is based on data which were systematically and prospectively recorded for every patient referred for the repair of ventral hernia combined with DR and that it draws attention on two issues that are not commonly addressed: 1) should the global bulging be included in DR assessment? 2) should IRD measured at rest, or on exertion, or both, be taken in consideration?

The main weakness is that CT scan measurements were available in 40 cases only, since they were not performed systematically in every case, and that some data are missing, such as measurement of the waist circumference in patients who had a global bulging, back pain evaluation, quality of life and body image assessment by questionnaires.

## Conclusion

Besides the median bulging, presence or absence of the global bulging should be included in DR assessment. Global bulging should be assessed by visual examination on the standing patient at rest, and ideally completed by measurement of the waist circumference just above iliac crests.

Besides measurement of the width of divarication at rest, measurement on exertion (leg-raise or curl-up) should be taken in consideration. The difference between measurements taken at rest and on exertion raises the question of which value should be used for diastasis classification. The question is worth being debated.

## Data Availability

The raw data supporting the conclusion of this article will be made available by the authors, without undue reservation.
